# Remarks on Residual Stress Measurement by Hole-Drilling and Electronic Speckle Pattern Interferometry

**DOI:** 10.1155/2014/487149

**Published:** 2014-07-06

**Authors:** Claudia Barile, Caterina Casavola, Giovanni Pappalettera, Carmine Pappalettere

**Affiliations:** Dipartimento di Meccanica, Matematica e Management, Politecnico di Bari, Viale Japigia 182, 70126 Bari, Italy

## Abstract

Hole drilling is the most widespread method for measuring residual stress. It is based on the principle that drilling a hole in the material causes a local stress relaxation; the initial residual stress can be calculated by measuring strain in correspondence with each drill depth. Recently optical techniques were introduced to measure strain; in this case, the accuracy of the final results depends, among other factors, on the proper choice of the area of analysis. Deformations are in fact analyzed within an annulus determined by two parameters: the internal and the external radius. In this paper, the influence of the choice of the area of analysis was analysed. A known stress field was introduced on a Ti grade 5 sample and then the stress was measured in correspondence with different values of the internal and the external radius of analysis; results were finally compared with the expected theoretical value.

## 1. Introduction

Almost every material presents, as results of production and manufacturing processes, an intrinsic field of stresses known in the literature as residual stresses. In some cases, they are also intentionally introduced in order to increase fatigue resistance and fracture strength. Due to the relevance of the topic, a number of techniques have been developed in order to measure residual stresses. X-ray diffraction can be used to measure interatomic strain caused by the presence of residual stresses (RS) introduced by different industrial processes such as those connected to shot peening [1] or friction stir welding [2]. This measurement allows calculating residual stresses once Young's modulus and Poisson coefficient are known. This kind of measurement is not destructive but in many cases it is limited to a few microns below the exposed surface. Penetration depth is increased up to tens of mm in systems using hard X-rays from synchrotron facilities [3]. Alternatively also diffraction of neutron beams can be exploited to determine residual stress as it was demonstrated on powder metallurgy component in [4] and in welded specimen in [5]. Both these systems are very expensive and difficult to implement.

Magnetic properties, such as Barkhausen noise, can also be exploited to evaluate residual stresses but only in ferromagnetic materials [6], however the Barkhausen noise depends on the hardness of the sample and for this reason it requires a calibration procedure in order to get quantitative results [7]. In the ultrasonic methods, the change in velocity of an ultrasonic wave propagating in the specimen can be related to the level of internal stress in the component [8]. Photoelasticity can be applied for measuring residual stresses in glasses [9]. Also Raman and fluorescence spectroscopic techniques have been applied to evaluate the presence of residual stresses [10]. Nowadays the hole-drilling method (HDM) is the most widespread one [11] and it is also ruled by a standard [12]. In this technique, residual stresses are calculated starting from the on-surface measured strains after drilling a hole in the specimen. This technique is semidestructive and nowadays there are some attempts to get stress relaxation in a nondestructive way by focusing a laser source on the sample to be analyzed to get local stress relaxation [13]. In hole-drilling method strain measurement is performed by strain gage rosette; this kind of procedure is quite reliable; however, it involves some drawbacks. For example, the surface of the sample needs to be prepared in order to attach the rosette and also the positioning of the drill bit must be accurately performed in order to avoid eccentricity errors [14]. Furthermore, only a few data points can be obtained by strain gage and this can introduce significant errors in the stress calculation procedure. Finally, it should be considered that the strain gage rosette has a cost, which cannot be considered negligible particularly in the case where a relevant number of measurements are required. Optical methods based on laser interferometry are promising in replacing strain gage in view of the fact that they can allow obtaining high sensitivity measurements from a huge number of data points without contact and allowing accurately defining, on the recorded image, the position of the drilled hole [15]. Moiré interferometry [16], phase shift shearography [17], and electronic laser speckle interferometry (ESPI) were successfully adopted in many different situations to measure displacement fields. ESPI was used to measure displacement field in anisotropic specimen made by selective laser melting [18], sinterization [19], or laminated wood [20]. Now ESPI is under study for the specific application in measuring residual stresses [21]. Specific studies were carried out in order to establish the effects of different parameters involved in the residual stress measurement process on the final accuracy. In [22] effects of several process parameters in RS measurement in titanium plates were investigated, while the effects of errors in the determination of the geometrical parameters characterizing the optical system are discussed in [23]. Finally, the effects connected to a proper choice of the drilling speed are reported in [24].

In this paper, the attention is focused on the phase shifting electronic speckle pattern interferometry used in combination with hole-drilling technique for measuring residual stress profile. This method, being an interferometric method, can guarantee sensitivity of the order of a fraction of the illumination wavelength, it does not require any particular preparation of the surface being applicable also to rough and curve surfaces, and it does not require application of a grating as in moiré interferometry [25]. The main drawback of this technique is the necessity to properly isolate the system from vibrations in order to obtain fringe pattern with good contrast.

Several works are present in the literature where ESPI has been used to measure strain and calculate residual stress. Focht and Schiffner used an ESPI set-up to measure strain in thin metal sheets [26]; a double beam ESPI interferometer was used by Baldi [27] to evaluate stresses in orthotropic materials; Díaz et al. measured by ESPI the stress relieved by hole drilling in an aluminum plate subjected to a uniform uniaxial tensile stress [28]; Viotti and Kaufmann evaluated the accuracy and the sensitivity of this hole-drilling and digital speckle pattern interferometry (DSPI) combined technique on aluminum thin plates subjected to a uniform uniaxial tensile stress [29] and described a portable device to measure residual stresses [30]; a mathematical method was proposed by Schajer and Steinzig for calculating residual stresses from hole-drilling electronic speckle pattern interferometry (ESPI) data, independent of rigid-body motions [31]; a method to cancel rigid body displacements that can be introduced when a hole-drilling and digital speckle pattern interferometry (DSPI) combined system is used to measure residual stresses was proposed by Dolinko and Kaufmann based on a least-square approach [32].

In this paper, some experimental considerations about error sources in the ESPI hole-drilling method will be discussed. In particular, the influence of the area of analysis on the final results in terms of measured residual stresses will be discussed. The inner and the outer radius of the area of analysis will be changed and the corresponding variations in the measured residual stress profile will be calculated.

## 2. Materials and Methods

### 2.1. Experimental Set-Up for HDM+ESPI Measurements

**Figure 1:**
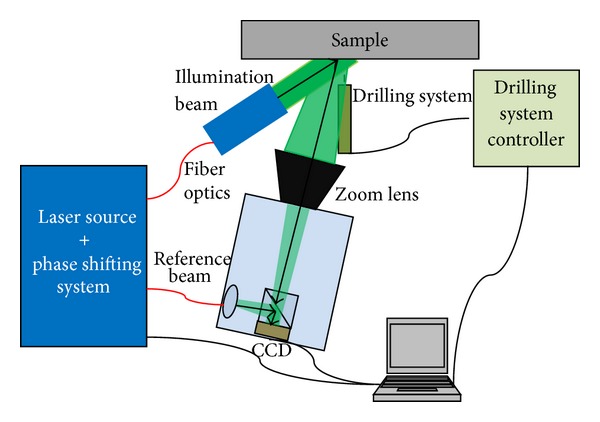
Experimental set-up for ESPI measurements of strains relaxed by HDM.

The ESPI hole-drilling measurement system used in this work is schematically reported in Figure 1. The laser source used in the proposed set-up was a diode-pumped solid-state (DPSS) laser source with a wavelength *λ* = 532 nm. Radiation was split into two beams and focused on two monomode optical fibers. One beam is collimated through a biconvex lens and illuminates the sample, while the second beam passes through a phase shifting piezoelectric system and then goes to a 640 × 480 pixel charge-coupled device (CCD) camera where interferes with the light diffused by the optically rough surface of the specimen. Camera is equipped with an optical imaging system allowing fine focusing of the image. The intensity recorded by the camera at each (*i*, *j*) pixel can be described by

(1) $$\begin{array}{c} I\left(i,j\right)=I_{\text{ref}}\left(i,j\right)+I_{\text{obj}}\left(i,j\right)+2\sqrt{I_{\text{ref}}I_{\text{obj}}}\cos\left(\varphi \left(i,j\right)\right), \end{array}$$

where *I*_ref_(*i*, *j*) is the intensity of the reference beam, *I*_obj_(*i*, *j*) is the intensity of the object beam deformations, and *φ*(*i*, *j*) is the initial phase difference.

If the surface of the specimen undergoes some displacement along direction of the sensitivity vector describing the system the speckle pattern experiences a phase difference Δ*φ*(*i*, *j*) and the new intensity $\overset{-}{I}\left(i,j\right)$ recorded by the CCD camera at each (*i*, *j*) pixel can be described by

(2) I−(i,j)=Iref(i,j)+Iobj(i,j)+2IrefIobjcos⁡⁡(φ(i,j)+Δφ(i,j)),

where Δ*φ*(*i*, *j*) is the additional phase difference connected with the displacement $\vec{d}$ experienced by the surface.

In order to extract the value of the additional phase the four-step phase shifting algorithm was implemented [33]. In order to do this in correspondence with the undeformed sample and for each stage of deformation of the specimen four images were recorded. A given image differs from the next one in view of the fact that piezoactuator is moved so that a known supplementary phase value equal to *π*/2 is added. Equations (1) and (2) can be rewritten as

(3) Ii(i,j)=Iref(i,j)+Iobj(i,j)+2IrefIobjcos⁡⁡(φ(i,j)+nπ2)Ii−(i,j)=Iref(i,j)+Iobj(i,j)+2IrefIobjcos⁡⁡(φ(i,j)+Δφ(i,j)+nπ2)

with  *n* varying from 1 to 4.

The additional phase Δ*φ*(*i*, *j*) can now be obtained by

(4) $$\begin{array}{c} \Delta \varphi \left(i,j\right)=\tan^{-1}\left(\frac{\left(I_{1}-I_{3}\right)\left(\overset{-}{I_{1}}-\overset{-}{I_{3}}\right)+\left(I_{2}-I_{4}\right)\left(\overset{-}{I_{2}}-\overset{-}{I_{4}}\right)}{\left(I_{1}-I_{3}\right)\left(\overset{-}{I_{2}}-\overset{-}{I_{4}}\right)-\left(I_{2}-I_{4}\right)\left(\overset{-}{I_{1}}-\overset{-}{I_{3}}\right)}\right) \end{array}$$

and finally displacement can be obtained by reminding the basic equation

(5) $$\begin{array}{c} \Delta \varphi =\vec{k}\cdot \vec{d}, \end{array}$$

where $\vec{k}$ is the sensitivity vector of the interferometric system.

The hole is drilled by means of an air-cooled high-speed turbine. Maximum rotation speed achievable with this turbine was 50000 rpm. The rotation of the turbine is electronically controlled so that it stays constant along all the drilling process. Experimental tests shown in this paper were all run at 50000 rpm in view of the fact that preliminary experiments displayed higher quality of the hole, for this material, at higher rotation speeds. In order to perform and control the displacement of the turbine it was mounted over a linear actuator having a travel of 25 mm and a resolution of about 0.05 *μ*m with bidirectional repeatability less than 1.5 *μ*m. The cutter is made by tungsten coated with TiCN and has a nominal diameter *d* = 1.59 mm.

Experimental measurements were performed on a titanium grade 5 specimen (248.5 mm × 42.5 mm × 3.0 mm). In order to generate a known stress field to be used as a reference to test the effect of using different analysis parameter the specimen was introduced in a four-point bending frame. The distance between the inner supports was 133 mm; the distance between the external supports was 208 mm. The distance between the centers of the holes was set to 12 mm to minimize the interaction between the holes. Holes were drilled at 12.0 mm from each edge in order to minimize edge effects. Before loading the sample, however, preliminary X-ray residual stress measurement was performed in order to evaluate that initial stress field on the specimen and a very low value of about 10 MPa was found. Subsequently, the HDM+ESPI method was utilized to confirm this “unloaded” stress field (the hole was drilled till 0.4 mm depth in the center of the specimen; each step was 0.05 mm). The same drilling parameters were also utilized for the subsequent tests on the loaded specimen. Concerning the calculation step *d*_calc_ = 0.1 mm was chosen in order to minimize the effects connected with random errors in strain [34, 35] and to better evidence effects connected with the choice of the area of analysis.

### 2.2. Considerations on the Area of Analysis

**Figure 2:**
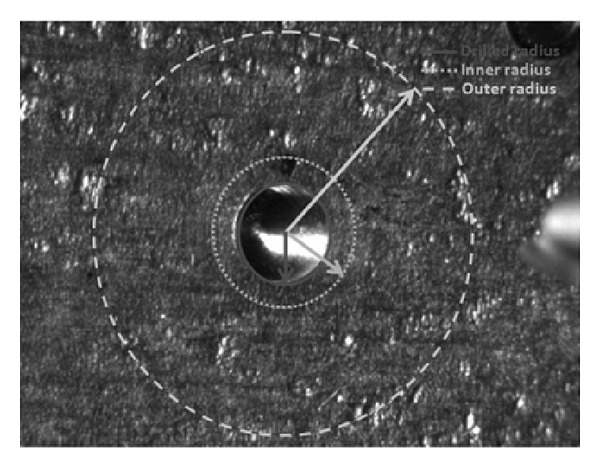
Screenshot of the area of analysis included between the outer circle (dashed line) and the inner circle (dotted line). The solid line identifies the drilled hole.

Strain measurements around the drilled hole were performed within an area delimited by two circles concentric with the drilled hole.  Figure 2 displays how the area of analysis is defined. The center of the area of analysis coincides with the center of the circle defining the edges of the drilled hole. Around this circumference two more concentric circles are defined. Pixels that will be effectively analyzed are only those included in the annular region delimited by those two circles.

The size of the analysis area can affect the results in terms of residual stresses. In fact, if the radius of the inner circle is too small, a region where plasticity effects occur is included in the calculation. Also particles generated during the drilling process can lay down around the holes. They constitute a noise for the interferometric measurement so that this occurrence can act as a further source of error. On the other side, if the outer radius is too large, a region of very small deformations can be included and this can lead to erring in the residual stress results.

In order to evaluate the influence of the analysis area the stress field was initially measured in correspondence with two fixed values of the inner radius (*R*_int⁡_) and of the outer radius (*R*_ext_), which delimitates the analysis area around the hole.

In order to highlight the influence of each variable on the obtained stress values, two situations were analyzed.

The first analysiswas performed by changing the inner radius in the range *R*_int⁡_ ∈ [1.11 mm, 1.59 mm] and keeping *R*_ext_ unchanged. Analogously, the outer radius ratio was changed in the range *R*_ext_ ∈ [2.07 mm, 2.62 mm] while keeping *R*_int⁡_ constant. The upper value 2.62 mm of the *R*_ext_ range was limited by the image dimension.

## 3. Results 

**Figure 3:**
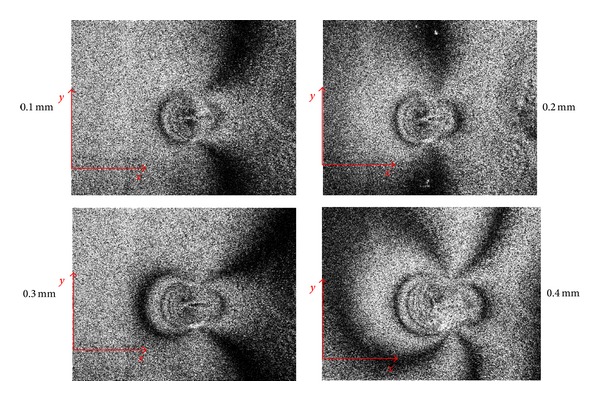
Speckle correlation fringes recorded after four drilling increments obtained by subtraction the real time recorded pattern from the initial reference pattern.

Recorded correlation fringe patterns obtained in correspondence with four different drilling steps are shown in Figure 3. Fringes are obtained by subtracting, from the reference speckle pattern recorded on the sample before starting the drilling process; the speckle pattern of the surface deformed as a consequence of the relaxed stress at each drill increment. Main qualitative features detectable in Figure 3 are the increment of the number of fringes in correspondence with higher drilled depths as a consequence of incremental stress relaxation and the presence of an axis of symmetry parallel to the *x*-axis which indicates the presence of an uniaxial state of stress.

**Figure 4:**
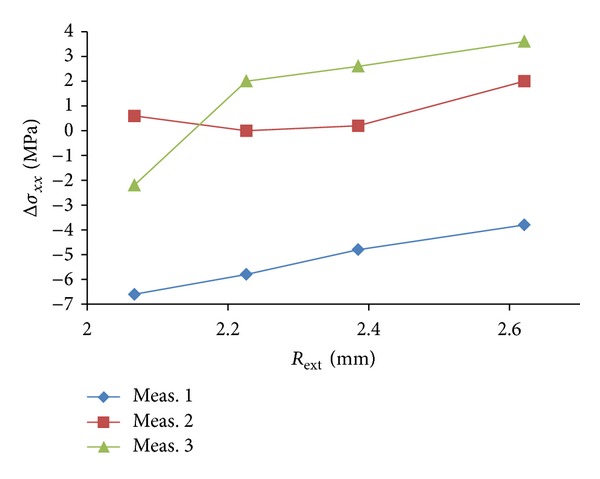
Plot of the average stress measured along the drilled depth in correspondence with different values of *R*_ext_ and by keeping constant the value of the internal radius ratio *R*_int⁡_ = 1.59 mm. The three curves refer to three different holes in the same specimen under the same stress conditions.

Also a reference system is reported: the *x*-axis is oriented along the longitudinal direction of the specimen and the *y*-axis is oriented along the transverse direction of the specimen.  Figure 4 shows the difference between the stress measured along the drilled depth and theoretical one, calculated by keeping constant the radius of the internal circle of analysis *R*_int⁡_ = 1.59 mm and by changing the value of *R*_ext_.

Table 1 reports the theoretical average stress values and the measured average stress values obtained by keeping constant *R*_int⁡_ = 1.59 mm and by changing *R*_ext_. Percentage errors are also indicated.

**Figure 5:**
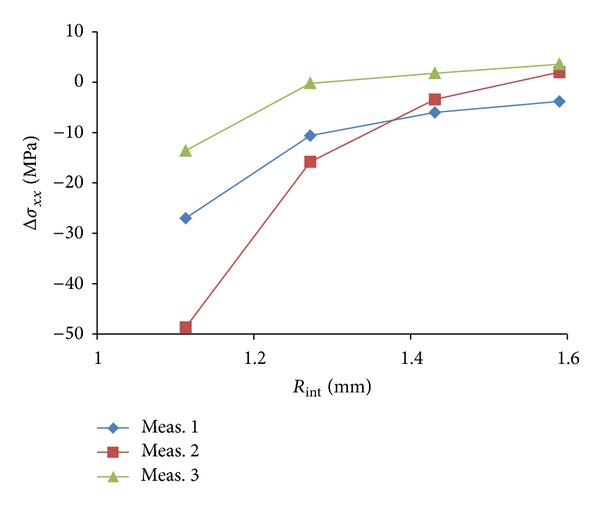
Plot of the average stress measured along the drilled depth in correspondence with different values *R*_int⁡_ and by keeping constant the value of the external radius ratio *R*_ext_ = 2.62 mm. The three curves refer to three different holes in the same specimen under the same stress conditions.

A further investigation on the analysis area was performed as follows: the radius ratio of the external circle of analysis *R*_ext_ = 2.62 mm was kept constant and the value of *R*_int⁡_ was changed (Figure 5).  Table 2 reports the theoretical average stress values and the measured average stress values obtained by keeping constant *R*_ext_ = 2.62 mm and by changing *R*_int⁡_. Percentage errors are also indicated.

In performing evaluation of the influence of the choice of the internal radius of analysis it was changed from *R*_int⁡_ = 1.11 mm up to the maximum value *R*_int⁡_ = 2.54 mm, which corresponds to a 56.3% of overall variation.

## 4. Discussion

It can be observed in Figure 4 that, by changing the external radius of analysis from *R*_ext_ = 2.07 mm up to the maximum value *R*_ext_ = 2.62 mm, which means a 21% of overall variation, the system shows an almost unchanged value of measured stress. In all cases, the found value is compatible with the expected theoretical value and no substantial influence of the set external radius on the final result can be evidenced.

As the analysis area expands by increasing *R*_ext_, the relaxation effect produced by the hole is less intensive at the outer; however, the presented results show that for the given material under analysis and for the given level of applied stress the ESPI system, used in this experiment, is able to accurately measure the value of deformation also in the outer region. As a consequence final results appear to be insensitive to the choice of the external radius.

Differently from what observed with changing the external radius, Figure 5 and Table 2 clearly show the influence of the internal radius on the final calculated stress value. While the measured stress value obtained by using *R*_int⁡_ = 1.59 mm is perfectly in agreement with the theoretical expected value, a significant discordance starts to be observed by using *R*_int⁡_ = 1.27 mm. In this situation, a 6.4% of difference is observed with respect to the theoretical value. Situation worsens even more if the internal radius is further reduced down to *R*_int⁡_ = 1.11 mm. Choosing this internal area of analysis the calculated average stress value is strongly different from the theoretical one and the discrepancy can be evaluated to be of the order of 21.6%.

The variation of the calculated stress with varying the internal radius of analysis can be justified in view of two aspects. First of all, it should be considered that plasticization effects could occur around the hole as a consequence of the drilling process. By varying the positioning of the internal radius *R*_int⁡_ it is possible to include, at a different extent, the plasticized region in the analysis area and this can affect the final result in terms of measured stress. Furthermore, it should also be taken into account that small particles are generated in proximity of the hole during the drilling process. A part of these flecks can remain attached to the surface especially in the surrounding of the border of the hole. The presence of these particles acts as a noise error for the electronic speckle pattern measurement. In fact, they can be considered like a local modification of the surface topology and they can also saturate the intensity of the corresponding image point. By reducing too much the internal radius of analysis, much of these particles can be included in the calculation and, as a consequence, the accuracy of the measurement is strongly reduced.

## 5. Conclusions

The influence of the variation of analysis parameters for residual stress measurement by hole drilling coupled with ESPI was carried out in this work. This is an important task in view of the fact that, differently from strain gage rosette where extensimeters are properly placed at a given distance from the drilled hole, in ESPI the choice of the pixel that must be included in the analysis is left to the operator. The study of the influence of the area of analysis has shown that a 24.2% variation of the external radius of analysis does not affect the final result. This means that, at least for the level of stress considered in this material, the ESPI system has enough sensitivity to properly detect strain independently from the distance where the outer area of analysis is placed. On the contrary, it was found that a 42.8% variation of the internal radius of analysis can introduce errors of up to about 21.6% on the measured stress. If compared with another analysis performed on the accuracy of the hole drilling + ESPI approach this appears to be a relevant aspect. In fact in the study of the effects of the drilling speed [24] it was found that it does not affect essentially the final results even if it can result in higher dispersion at low velocity (up to 20%) due to the poor quality of the drilled hole in that condition. The study of the errors introduced by a bad determination of the geometrical parameters [23] indicates that the most critical parameters, that is to say, the angle between the optical axis of the camera and the normal to the measurement surface, can introduce errors up to 5%. This occurrence suggests that attention should be paid to the proper choice of the internal radius of analysis being a critical parameter that can severely affect the quality of the measurement. The reason of the influence of this parameter can be tracked back mainly to two effects: the plasticization that occurs near to the hole due to both the stress concentration generated by the drilled hole and by the drilling operation itself and also the possible presence near the edges of the hole of small metallic particles that alter the surface and that can, as a consequence, introduce errors in the phase calculation from the speckle pattern. This last effect could be reduced by implementing proper measurement procedures which include dust removal from the surface at the end of each drill increment. Air compress can be used for this scope. Attention should however be paid to not moving the sample during this operation in order to not introduce any fictitious displacement. Also care should be paid to not stain the surface with any impurity that could be present in the compress air. It could be interesting, as a future work, to explore the extension of the plasticization region by performing measurements at different drilling rotation speeds in order to change the thermal input to the specimen during the drilling process.

## Figures and Tables

**Table 1:** Values of the measured average stress obtained by keeping constant *R*_int⁡_ = 1.59 mm and by changing *R*_ext_. The expected theoretical value was *σ*_*xx*_ = 138 MPa. Also standard deviations are reported as a result of three different drilled holes.

	*R*_ext_ = 2.07 mm	*R*_ext_ = 2.23 mm	*R*_ext_ = 2.38 mm	*R*_ext_ = 2.62 mm
*σ*_*xx*_ [MPa]	135.3	136.7	137.3	137.3
|Δ*σ*_*xx*_| (%)	1.9	0.9	0.5	0.4

**Table 2:** Values of the measured average stress obtained by keeping constant *R*_ext_ = 2.62 mm and by changing *R*_int⁡_. The expected theoretical value was *σ*_*xx*_ = 138 MPa. Also standard deviations are reported as a result of three different drilled holes.

	*R*_int⁡_ = 1.11 mm	*R*_int⁡_ = 1.27 mm	*R*_int⁡_ = 1.43 mm	*R*_int⁡_ = 1.59 mm
*σ*_*xx*_ [MPa]	108.2	129.1	135.5	138.6
|Δ*σ*_*xx*_| (%)	21.6	6.4	1.8	0.4
